# Data Glove Using Soft and Stretchable Piezoresistive Sensors

**DOI:** 10.3390/mi13030372

**Published:** 2022-02-26

**Authors:** Kean Aw, Jessica Budd, Thomas Wilshaw-Sparkes

**Affiliations:** Department of Mechanical and Mechatronics Engineering, The University of Auckland, 1010 Auckland, New Zealand; jbud314@aucklanduni.ac.nz (J.B.); twil459@aucklanduni.ac.nz (T.W.-S.)

**Keywords:** stretch sensor, piezoresistive, data glove

## Abstract

This research investigates the design and implementation of elastomer-based piezoresistive strain sensors and applies them to a data glove to demonstrate their application. The piezoresistive strain sensors are made by mixing Ecoflex 00-30 and carbon-black nanoparticles and then using stencil and doctor blading to deposit the piezoresistive traces as a mass fabrication technique. The primary objective is to integrate two sensing piezoresistive elements as one single-piece sensor that detects the bending angles of the metacarpophalangeal and proximal interphalangeal joints of each finger. Using a unique zig-zag pattern allows to selectively mask any unwanted piezoresistive sensing. The sensor has a gage factor of 0.68. Experiments conducted have demonstrated that the use of these soft, flexible, and stretchable piezoresistive sensors is repeatable and viable sensors for data-glove and has the potential for other wearable applications.

## 1. Introduction

There has been increasing demand for lightweight, low-profile mechanical sensors for use in soft robotics and wearable devices, such as prosthetics and exoskeletons. Soft stretchable sensors can be used to determine the angular displacement of joints while their compliance and flexibility minimize their impedance on the motion. Resistance changes are used to indicate strain levels, which can be used to measure the extent of joint movement.

There are many different applications for strain sensors in wearable devices where joint motion is to be measured, including industrial, medical and entertainment uses. Humans wearing data gloves could remotely control a mechanical hand or gripper in hazardous environments such as outer space or in factories to handle hazardous materials or machinery [1,2]. In the medical field, wearable strain sensing devices allow surgeons to perform operations remotely, requiring an extremely high level of accuracy and usability. Similarly, they could allow students to practice surgeries in a simulated environment, reducing the need for cadavers or live animals [3]. Wearable strain sensing devices could also allow digitization of hand motion, helping diagnose, prescribe treatment, and monitor rehabilitation progress of motor affecting diseases [4].

Data gloves and wearable devices are already widely used in virtual reality, particularly in the rapidly expanding video gaming industry. Combining these with haptic feedback has also shown potential in teaching musical instruments, such as with virtual pianos [5]. Furthermore, innovations such as the Mi.Mu gloves or similar gloves developed by [6] are themselves musical instruments, using hand gestures to control musical elements. Motion capture is already widely used in the film industry when creating computer-generated imagery. This often uses several cameras tracking markers on the actors but struggles to fully capture complex hand motion [7]. There is potential here to accurately capture hand gestures and orientation at the source using soft strain sensors.

Data gloves have already been used in recording sign language for communication between deaf people and those who do not understand sign language [8]. The gloves are worn by deaf or mute persons communicating via sign language, which is then translated to spoken or written language for the receiver. This allows these people to have effective communication with the whole population and has already been successful enough to be carried out in several languages.

The sensors this study aims to develop could prove advantageous in machine and robotics control. Similar to a data glove, a stretchable strain sensor can be placed over a moving or sliding joint to provide position data to a controller. Their low profile, non-obstructive, and lightweight properties could negate the need for heavy encoders to provide feedback on each joint’s position, simplifying mechanical structures. Such sensors could be particularly useful in the field of soft robotics where there are generally no discrete joints actuated by single motors, instead of being actuated by pneumatics, electric or magnetic fields, or other bio-inspired methods [9]. This field has seen increased research in recent years with much focus on safe robot-human interaction, soft handling of unpredictable and delicate objects, and low energy consumption. Considering the dynamic nature of these devices, a soft stretchable strain sensor capable of measuring strain in multiple regions could be highly applicable at providing live position feedback.

Given the extremely high dexterity of the human hand and its ability to manipulate its environment, it is desirable to grant this capability to robots and other autonomous machines. Data gloves could allow hand motion and gestures to be recorded digitally. Data gloves provide the ability to record a variety of human hand motions which can then be learned by fully autonomous robots so they may interact with their environment without requiring live human input, using soft stretchable strains sensors to acquire real-time feedback on their motion [10]. This article aims to mainly demonstrate the unique design of a large strain piezoresistive sensor that allows the sensing of the bending angles of the metacarpophalangeal and proximal interphalangeal joints of each finger. The use of a unique zig-zag piezoresistive pattern allows to selectively mask any unwanted piezoresistive sensing and allows the integration of two sensors into a single piece. The application of this sensor onto a data glove is to demonstrate the application of this unique design.

## 2. Soft and Stretchable Piezoresistive Sensor

**Figure 1:**
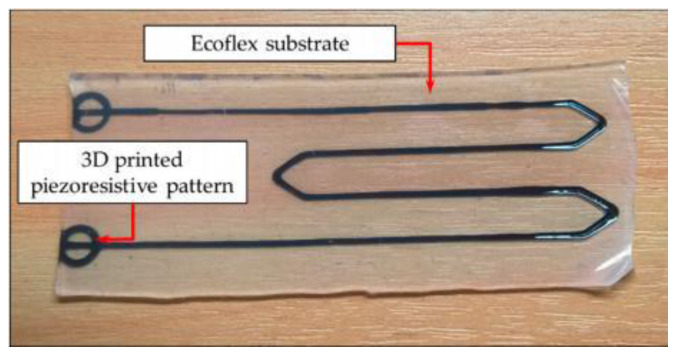
A large strain piezoresistive (50 mm in length) made from printed carbon-black and Ecoflex 00-30 paste [12]. The two circles on the left-hand side are the electrical connections to the sensor.

There are several different ways to use piezoresistance for strain measurement, including elastic conductive fabric, soft stretchable conductive materials, and conductive liquids [11]. Soft stretchable piezo-resistive sensors often use a thin layer of conductive material mixed with an elastomer to form a stretching conductive element which is then encased within two more layers of elastomer. The elastic conductive piezoresistive element in the form of paste can be printed in any design on a 2D plane, such as the carbon nanotubes/EcoFlex based sensor from our previous work [12] shown in Figure 1, making them relatively compact.

By using a carbon black-EcoFlex mix as a paste to form a stretchable conductive element, such as in Figure 1, a single-piece sensor capable of measuring strain at two finger joints independently should be possible. The highly elastic and bending properties of the material, along with the compact nature of the conductive element should enable two sensors to be equally compact and not reduce dexterity. These sensors also have an approximately linear relationship between strain and change in resistance making their outputs very easy to digitize [11]. Their material cost is low relative to other sensor types, and they are easy to manufacture. The circuitry required is simple and is undemanding on space.

Disadvantages to piezo-resistive strain sensors do exist, however small they are. Piezo-resistive strain sensors experience some influence from changing temperatures. However, this is negligible for this study’s applications and can be remedied with recalibration as the strain versus resistance relationship is not affected by temperature. Our previous experimental results [11] have shown that optimal designs suffer from hysteresis error of approximately 10%. Given this study does not aim to produce high accuracy results, this hysteresis is practically acceptable.

Elastomers can suffer stress relaxation over prolonged periods of strain and many thousands of stretch cycles. While this is worth considering, studies into EcoFlex 00-30 found virtually no decrease in nominal stress when held under 200% strain for 3 h, negating any concerns [13], and in the application as data glove a linear strain of not more than 30% is sufficient.

**Figure 2:**
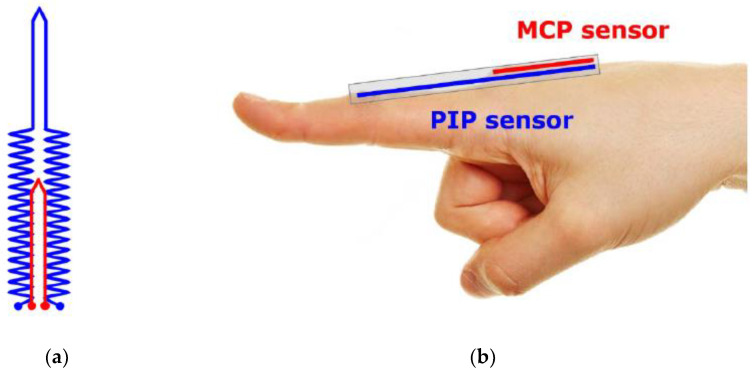
(**a**) Top view of the two-layer sensor design, (**b**) the placement of the sensor to measure the bending of the PIP and MCP joints.

**Figure 3:**
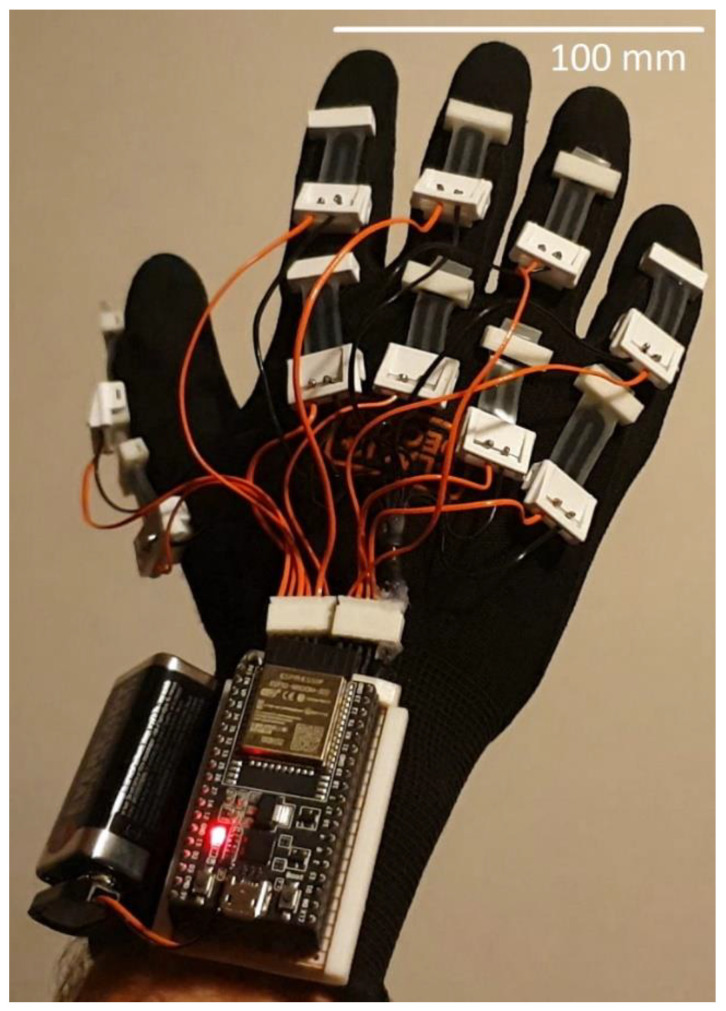
A data glove with an individual sensor for each joint. Note the wiring to each sensor.

While the printing technique is attractive it is not suitable for mass fabrication. In this article, a mass fabrication technique using stencil and doctor-blading of the carbon-black/Ecoflex paste on an Ecoflex substrate will be used. Details of the fabrication technique will be presented in the later section. A two-layer integrated sensor design contains two stretch elements: one for the proximal interphalangeal (PIP) joint and another for the metacarpophalangeal (MCP) joint is proposed (see Figure 2a). The sensing element consisted of the conductive path made from a mixture of carbon black and Ecoflex, in line with the direction of strain for maximum change in resistance. An illustration of this sensor placement with respect to a hand is shown in Figure 2b. From Figure 2, it is clear that the sensor’s carbon-black/Ecoflex trace that aims to only measures the PIP joint also runs through the MCP joint. Hence, the bending of the MCP and PIP will both be picked up if a simple V-shape trace is used. The use of a unique zig-zag trace at the location of the MCP joint (See blue color trace in Figure 2) will mask any change in resistance if the MCP joint bends. Stacking two sensing elements in the configuration, shown in Figure 2, allows the integration into one single piece. Using this integrated configuration allows all electrical connections to be made at the same edge, otherwise, two separate sensors will be required to measure the MCP and PIP joints with untidy wiring, as shown in Figure 3.

Referring to Figure 2, the top sensing element (red color) is meant to only measure the bending of the MCP joint and will be termed the MCP sensing element. The MCP sensing element (red color) consists of only a V-shape section where the conductive trace will stretch when the MCP joint bends. However, the PIP sensing element (blue color) consists of 2 sections, i.e., the zigzag and the V-shape sections. The V-shape section coincides with the PIP joint as it is aimed at only measuring the bending of this joint. As in Figure 2, the bottom sensing element (blue color) has a unique zig-zag trace at the MCP joint and V-shape at the PIP joint is meant to only measure the bending of the PIP joint and hence will be termed as the PIP sensing element. The design is based on the understanding that there will be little change in the resistance when the MCP joint bends as the actual elongation of the zig-zag trace will be very limited. However, as the V-shape section lies at the PIP joint, any bending of that joint will cause the V-section to stretch and hence increase the resistance. Using this unique design, the MCP and PIP sensing elements can be integrated into one single piece instead of two separate pieces (see Figure 3), making the integration with the data glove more seamless as it will be easier to make electrical connections to the electronics.

## 3. Sensor Fabrication

This section describes the fabrication of sensors using stencil and doctor-blading in contrast to our earlier printing technique. This technique allows mass fabrication of sensors. The first step of the sensor fabrication was to create an initial layer of Ecoflex 00-30 within a mold. Acrylic frames, held together by metal binder clips, were used as a mold for the Ecoflex layers. These frames were large enough to contain at least eight full-length sensors and up to 16 single joint sensors at once.

The thickness of the initial layer was 0.6 mm thick Ecoflex cured in an oven for an hour at 70 °C.

**Figure 4:**
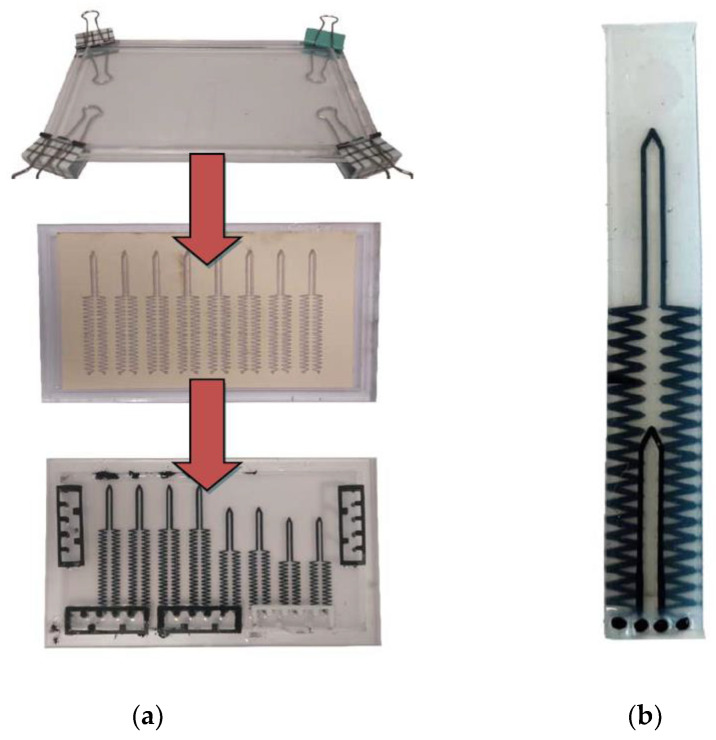
(**a**) Stages of fabricating sensor, (**b**) completed double-layer sensor.

When cured, a trace of carbon black/Ecoflex paste is applied using a stencil for the PIP sensing element. The carbon black was purchased from The Fuel Cell Store, USA, and has a particle size of 50 nm. The carbon black/Ecoflex paste is made by thoroughly mixing a weight ratio of 1:9 carbon black to Ecoflex. This ratio was found to produce suitable piezoresistive behavior, as reported in [11]. The carbon black/Ecoflex paste is applied onto the paper stencil and then doctor bladed before placing in the oven for 24 h at 70 °C. Next, another insulating Ecoflex layer of 0.8 mm was made on top of the PIP sensor. Then carbon black/Ecoflex paste was doctor bladed using a stencil to make the MCP sensing element. Finally, the top layer of Ecoflex, with a thickness of 0.6 mm protects the MCP sensing element. This process is shown in Figure 4a. The completed double layer sensors were cut to size. Figure 4b shows a completed double layer integrated sensor that consists of the MCP and PIP sensors cut to size and each is required for each finger.

## 4. Sensor Characterization

**Figure 5:**
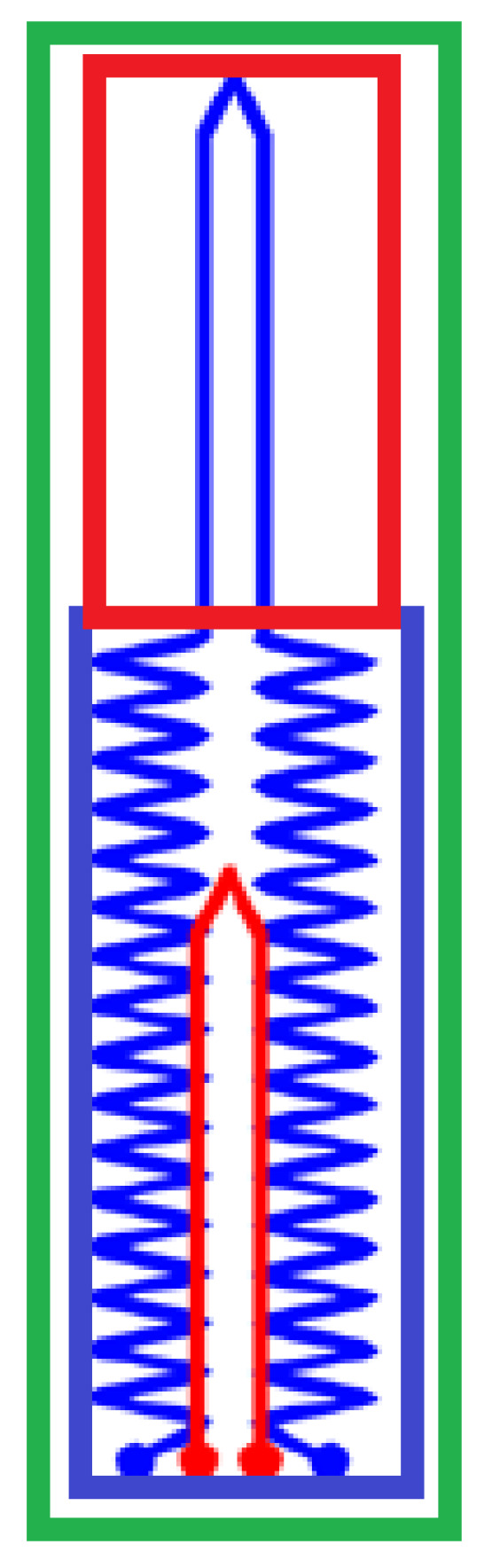
A double layer stretch sensor, where the PIP sensing element (green) is divided into the zigzag and V-shape sections.

The sensor is characterized using a rig where the sensor is stretched, and the resistance is measured with an LCR meter (Agilent 4263B). The PIP sensing element is characterized. Initially, the whole PIP sensing element (V and zigzag sections) is characterized (green in Figure 4) by clamping at the two extreme ends, and then the resistance is measured as it is stretched. This will produce the results where the V-shape and zig-zag sections will be stretched simultaneously demonstrating that the MCP and PIP joints are simultaneously bent. Another characterization only involves just clamping the sensors so that only the V-shape section of the PIP sensing element (red in Figure 5) is stretched, demonstrating only the PIP joint is bent.

To further confirm that the zig-zag section does not change in resistance when the MCP joint bends, the sensor was clamped in such a manner that only the zig-zag section is stretched and the resistance is recorded. The sensor is stretched up to a linear strain of 30% as this is sufficient to determine the joint angle when the fist is fully clenched. The gage factor was calculated to be 0.68 based on the assumption of a linear relationship between the resistance and the strain.

**Figure 6:**
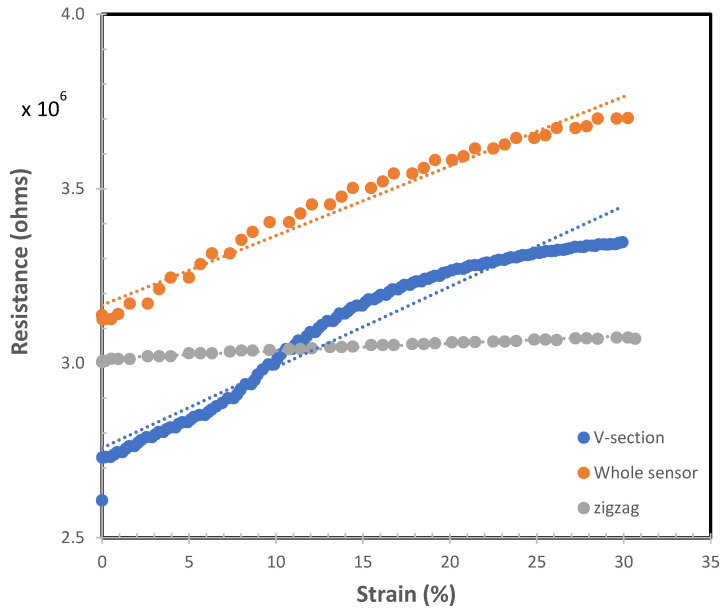
The resistance versus linear strain of the PIP sensing element shows the relationship of the V-section, zig-zag section, and the whole sensor.

From Figure 6, it is clear that the zigzag section of the PIP sensing element contributes insignificant change in resistance when stretched. This validates that the PIP sensing element with the V-shape and zig-zag sections (bottom blue sensor in Figure 2a) will only change in resistance when the PIP joint bends and will not cause a significant change in resistance when the MCP joint bends. This proves that the use of a unique zig-zag pattern can mask any unwanted piezoresistive effect. The bending of the MCP joint will only be measured by the MCP sensing element that was fabricated on the top layer (red sensor in Figure 2a) and has similar resistance versus strain relationship as the V-section of the PIP sensing element (see Figure 6). The gauge factor was calculated to be 0.68 based on the assumption of a linear relationship between the resistance and the strain.

## 5. Data Glove

**Figure 7:**
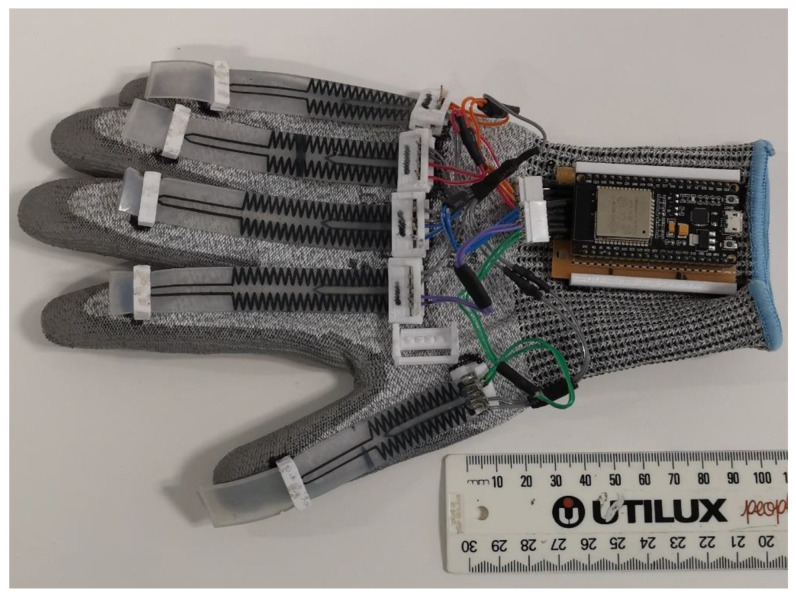
A completed data glove showing the stretch sensors connected to an Arduino board with a Bluetooth shield.

Five sensors using this unique configuration were made and attached to each finger of a glove. Each sensor has four terminals, two each for the PIP and MCP sensing elements. Each sensor is considered a variable resistance in a potential divider circuit. As the resistance of the sensor changes the voltage of the potential divider changes. The voltage represents the bending of the PIP or MCP joint. The voltage is then converted into the equivalent digital value in the Arduino and will be wirelessly transmitted via Bluetooth to a computer. The computer will present the hand gesture graphically. Figure 7 shows the completed data glove.

**Figure 8:**
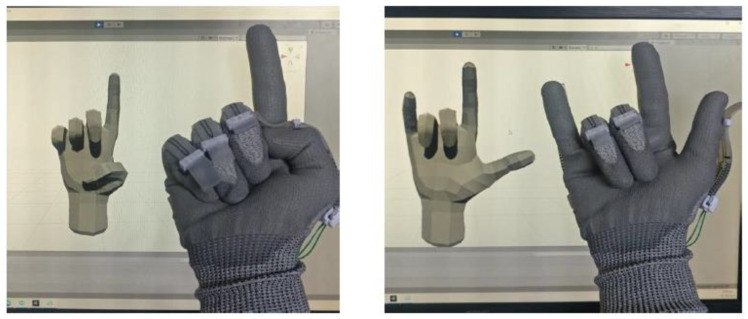
Examples of hand gestures and their presentation on the computer screen.

The Unity game engine is used to model the hand on the computer. The PIP and MCP joints of each finger are determined by the sensors. The conversion from the resistance to joint angles is performed by Arduino. The joint angles for each finger are then transmitted via Bluetooth to the computer where the hand gesture will be graphically displayed via the Unity game engine, as shown in Figure 8.

## 6. Conclusions

A simple low-cost piezoresistive stretch sensor based on carbon black and Ecoflex mixture has been successfully fabricated using stencil and doctor-blading for mass fabrication. Each sensor has two layers of carbon black/Ecoflex trace to measure the angles of the proximal interphalangeal and metacarpophalangeal joints. Using this unique zig-zag pattern allows the masking of piezoresistive effect by the bending of a specific joint. The implementation of five pieces of this integrated configuration onto a glove demonstrates the application of this sensor. In this prototype, the bending angle of the distal interphalangeal (DIP) joint is not measured as in most cases this joint is coupled with the proximal interphalangeal joint. However, a tri-layer sensor can be fabricated using a similar scheme of using a unique zig-zag trace to incorporate this additional DIP joint. Despite the low gauge factor of 0.68, it was demonstrated to be sufficient to be implemented into a data glove. There are some limitations, as observed from the supplementary Video S1, where sometimes there are some slight discrepancies between the actual gestures and the represented gestures on the computer screen. This is due to the inherent behavior of elastomer-based strain sensors, such as stress relaxation and variation due to different strain rates, as reported in our previous work [11,12].

## Supplementary Materials

The following supporting information can be downloaded at: https://www.mdpi.com/article/10.3390/mi13030372/s1, Video S1: micromachines-13-00372-supplementary.mp4.
